# Image Processing Algorithm for In Situ Monitoring Fiber Laser Remote Cutting by a High-Speed Camera

**DOI:** 10.3390/s22082863

**Published:** 2022-04-08

**Authors:** Max Schleier, Benedikt Adelmann, Cemal Esen, Ralf Hellmann

**Affiliations:** 1 Applied Laser and Photonics Group, University of Applied Science Aschaffenburg, 63743 Aschaffenburg, Germany; benedikt.adelmann@th-ab.de (B.A.); ralf.hellmann@th-ab.de (R.H.); 2 Applied Laser Technologies, Ruhr-University Bochum, Universitätsstraße 150, 44801 Bochum, Germany; esen@lat.rub.de

**Keywords:** laser remote cutting, high-speed camera, image processing

## Abstract

We present an in situ process monitoring approach for remote fiber laser cutting, which is based on evaluating images from a high-speed camera. A specifically designed image processing algorithm allows the distinction between complete and incomplete cuts by analyzing spectral and geometric information of the melt pool from the captured images of the high-speed camera. The camera-based monitoring system itself is fit to a conventional laser deflection unit for use with high-power fiber lasers, with the optical detection path being coaxially aligned to the incident laser. Without external illumination, the radiation of the melt from the process zone is recorded in the visible spectral range from the top view and spatially and temporally resolved. The melt pool size and emitted sparks are evaluated in dependence of machining parameters such as feed rate, cycles, and focus position during cutting electrical sheets.

## 1. Introduction

Remote laser cutting, typically using laser scanning systems rather than conventional laser optical cutting heads with a coaxial gas stream, is well established in industry for cutting thin metal sheets. During remote laser cutting, the metal is heated locally to temperatures above the boiling point, and melt is ejected due to the vapor and recoil pressure of the boiling metal [1,2]. During this process, liquid melt particles are ejected from the process zone as sparks [3]. Unlike conventional laser fusion cutting, where the material molten by the laser beam is expelled downward in the kerf with an inert gas, during remote laser cutting, the sparks, vapor, and melt ejection are blown away sideward with a crossjet to protect the optics. For remote cutting, the material removal rate during one ablation cycle depends on the material, laser power, and feed rate [4], and it can generally be determined to about 30 µm in depth [5,6]. By repeating the scanning step in a multi-pass processing approach, a complete cut through the material is achieved. Remote laser processing has been comprehensively studied and is widely used [1,2,3,4,5,6]. However, challenges remain for realizing a reliable monitoring system for in situ quality control. 

The duration and number of successive cycles have a significant influence on the remote cut quality. During cutting in a multi-pass approach, the metal sheet tends to slightly warp due to thermal input. Thus, during any subsequent cycle, the impinging laser may also radiate onto the sheet offset where the intended kerf is located. This increases inaccuracy and creates additional burr. Furthermore, for lower feed rates, welding of the kerf may occur. Since the workpiece cannot be detached from the sheet due to the welding, it can neither fall down into the flat bed nor be detached by a pick-and-place machine. This usually leads to the exclusion of the workpiece and the sheet. Therefore, an important requirement for a quality controlling system is the ability to observe the spatially and temporally resolved process zone, which, in turn, requires an imaging sensor with a high sampling rate. 

In contrast to selective laser melting and laser fusion welding, for which different sensing concepts have already been reported [7,8,9,10,11,12,13], a monitoring setup for remote laser cutting, especially for near-infrared lasers, is barely reported or in industrial use. For most selective laser melting applications, similar laser scanning heads are used as for remote laser cutting. Quality monitoring systems, such as high-speed cameras with an external illumination source due to high scanning speeds [14] or a combination of an IR camera and a photodiode [15], detect the melt pool emission of the powder bed during the melting process. 

Different image sensor-based approaches have been reported, for instance, from a coaxial perspective for CO_2_ laser cutting with additional illumination [16,17] and without external illumination [18], where the thermal process radiation is measured, while the reflected primary laser radiation is filtered. For NIR lasers, approaches have been reported for in situ detection via a trim-cut technique through a transparent substrate for a clear visualization inside the cutting kerf to monitor and measure the melt flow dynamics on the cutting front [19,20,21] and using a side visual monitoring setup to capture the melt ejection [22]. Initial approaches to monitor the process zone coaxial for fiber laser flame cutting [23] and fiber laser fusion cutting [24] have also been reported.

Monitoring approaches with a high-speed camera have been reported to detect the formation of spatter for laser welding [25,26]. As an alternative to quality monitoring with cameras, sensor systems based on photodiodes have also been developed to measure the quality for laser cutting [27,28] and selective laser melting [29,30]. However, geometric information about the process area is relevant for reliable monitoring. In addition to sensor technology and process detection, an evaluation algorithm is essential for quality monitoring, especially for industrial applications. A cross-correlation algorithm [31] and a polynomial logistic regression model [32] have been reported to detect and predict cut interruptions. Computer vision, which analyzes and processes images captured by cameras, and neural networks, as a subfield of machine learning, has been successfully used for image classification and recognition of geometric information from digital images, such as object and face detection [33,34,35], in medicine and biology for X-ray [36] and electron microscope images [37], and for laser fusion cutting [38,39]. A recent report proposed a neural network to predict the kerf width for laser remote cutting calculated from the input parameters of laser power, feed rate, and pulse frequency [40].

Against this comprehensive background, in this paper, we demonstrate an in situ monitoring system based on a high-speed camera (HSC) for remote fiber laser cutting of electrical sheets. The sensor system can be attached to a conventional remote cutting head for fiber lasers. This setup allows us to directly view the emitted secondary thermal process radiation from the process zone from the top view with a high frame rate in the visible spectral range. 

## 2. Materials and Methods

### 2.1. Laser Cutting Experiments 

**Figure 1:**
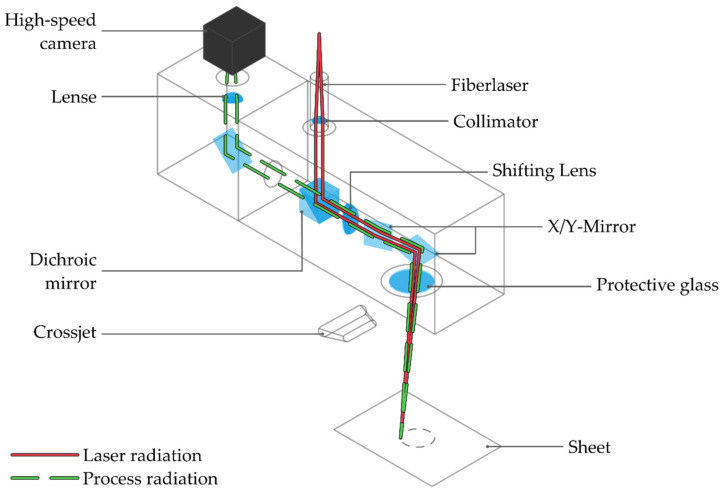
Schematic illustration of the monitoring system and scan head.

A series of experiments on laser remote cutting were performed with a high-power capable scanner (Fiber Elephant, Novanta Europe GmbH) along with a 1 kW multimode fiber laser with an emission wavelength λ of 1064 nm, a beam quality factor M^2^ of 1.51, and a focal point diameter of 45 µm. The setup of the laser scanning system with the HSC is shown in Figure 1. The primary laser beam from the beam source, represented by a red line, was collimated, reflected at a dichroic mirror, and guided onto a galvanometer scanner. The laser beam was deflected over the workpiece surface by two rotatable scan mirrors for the *X-* and *Y*-positions. A process lens was used as focusing element, which also corrected the focus position shift in the scan field. 

The thermal process radiation from the process zone propagated omnidirectionally from the process zone, partly in the direction of the scan head, and it was reflected at the galvanometer mirrors as depicted by the green dashed line in Figure 1. The reflected radiation was separated optically by a dichroic mirror, whose dielectric coating was reflective for the emission wavelength of the laser. In turn, the wavelength range of the visible thermal radiation from the process zone was transmitted and guided to the HSC.

The aim of this setup was to measure the thermal radiation from the process zone during the cutting process in situ from the perspective of the top view through the scan head in the VIS spectrum. Remote cutting was performed on electrical sheets type M270 at sheet thicknesses of 0.35 mm and 0.50 mm, as these are the most commonly used for electric motors due to low eddy currents and high productivity. Productivity per stack increases for thicker sheets, but this also increases eddy currents and, thus, impairs motor performance. For thinner sheets, the production costs per stack increase, and, due to their lower stiffness, it is more difficult to cut such thinner sheets. 

A good cut in which the workpiece can be easily detached from the sheet or falls out by itself is achieved with a feed rate of 7 m/s with 10 cycles and a laser power of 1 kW, with the focus position on the sheet surface for sheet thicknesses of 0.35 mm. For the experimental design of this study and for the evaluation of the designed monitoring approach, these parameters were varied. The laser power was not varied and set to 1 kW for all experiments. The feed rate was limited to a maximum of 7 m/s by the scanning head; hence, the feed rate was varied from 1 m/s to 7 m/s. The number of cycles was varied between five and 100, and the focus position of the laser was varied between −3 mm and +3 mm. Incomplete cuts were obtained by a reduced number of cycles. Rewelding was forced by a reduced feed rate or by a focus shift. Incomplete cuts due to thermal stress were enforced by a lower feed rate or by an exceeding number of cycles. 

**Figure 2:**
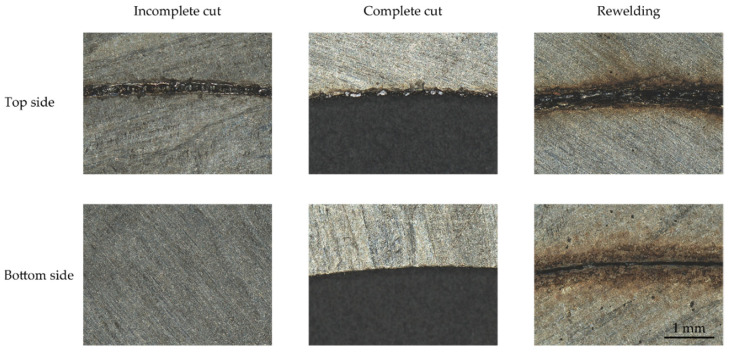
Exemplifying images of the top and bottom side of the electrical sheets for an incomplete, complete, and rewelded cut.

The selected cutting contour for this study was a circle with a circumference of 120 mm. A circle was chosen to be independent on the feed rate direction and based on elliptical or circular cutout shapes of armature or stator of electric motors. Exemplifying images of the top and bottom side of the electrical sheets for rewelded, incomplete, and complete cuts are presented in Figure 2. For a complete cut, a clean edge can be seen on the top and bottom surfaces. Typical for remote laser cutting, burr formation can be observed on the top side of the sheet instead of the bottom edge, since the melt ejection is directed upward [2,41]. For incomplete cuts, the top of the sheet shows a melted path. The bottom side of the sheet appears unprocessed, with no heat input or material removal visible. For rewelded cuts, a kerf can be seen on the top and bottom surface, but these are rejoined due to burr formation or welding in the kerf. In addition, a large heat-affected zone can be seen.

### 2.2. Monitoring Setup 

**Figure 3:**
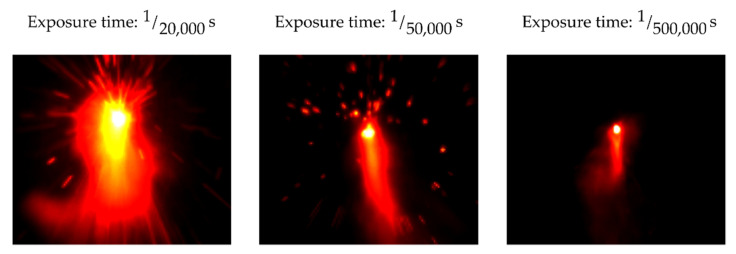
Comparison between different camera exposure times with the same cutting parameters.

The camera, a Photron Fastcam Mini AX50, contained a CMOS chip with a square pixel size of 20 × 20 µm^2^, a relative spectral sensitivity between 400 and 700 nm, and an onboard ring buffer, where the videos were stored before subsequent downloading. To visualize the thermal radiation, the process zone was recorded at a sampling rate of 20 × 10^3^ frames per second. The maximum image size depends on the variable sampling rate and decreases for higher sample rates. At the chosen sampling rate of 20 × 10^3^ fps, a maximum image size of 256 × 256 pixels was possible. The exposure time was set to 20 µs (1/50 k s). Figure 3 shows the same cutting process with different exposure times. For shorter exposure times, insufficient process radiation reached the scan head, whereas, for longer exposure times, the image was overexposed. This, however, is favorable to observe flying sparks, as shown in Figure 3.

At the maximum exposure time, limited by the sampling rate of 50 µs (1/20 k s), sparks were seen as long light trails and a blurred melt pool. For a shorter exposure time of, for example, 2 µs (1/500 k s), only process radiation directly from the melt pool and no sparks were observed. With the employed exposure time of 20 µs, both melt and flying sparks could be recorded. The thermal radiation from the process zone was recorded without additional illumination. To focus the melt pool, the camera focus was set equal to the laser focus. For the optical output, a C-mount camera connector was attached to the scan head.

### 2.3. Evaluation of the Images

**Figure 4:**
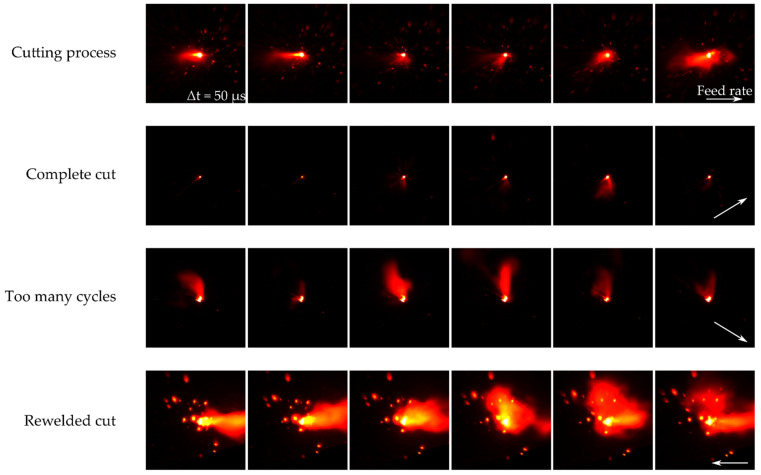
Image samples for different cutting qualities.

To monitor the quality of laser remote cutting, spectral and geometric information from the secondary process radiation in the process zone was evaluated in the VIS by an image processing step. The aim was to monitor the complete cut after the cutting process before the occurrence of thermal distortion due to the heat input from the laser radiation on account of too many cycles. Additionally, welding processes were detected due to possible feed rate fluctuations. Figure 4 shows exemplifying images for the cut categories during the cutting process, i.e., a complete cut, too many cycles, and a reweld of the workpiece to the sheet. During the cutting process, the melt in the center of the process zone emitted more intensely and more in the green and blue spectral range due to the higher temperatures. Presumably due to overexposure and additive color merging of the RGB channels, the melt in the kerf glowed yellow or white in the captured image. The vapor plume formation emitted primarily in the red spectral range and was, therefore, presumably cooler than the melt pool. Once a complete cut through occurred, the melt pool size decreased significantly as the kerf was opened downward through the sheet. This led to a smaller contact area between the laser radiation and the processed material.

As a result, the absorption of the laser radiation was reduced. Thus, the intensity of the process radiation decreased. The number of sparks and the size of the metal vapor decreased significantly and occurred only sporadically or were expelled below the sheet. If too many cycles were performed, the meld pool size increased again slightly as the intended kerf shifted. Due to thermal input or displacement of the workpiece, the primary laser could radiate again onto the sheet or workpiece, resulting in plume formation. If rewelding occurred, the kerf rejoined and the size of the melt pool, plume formation, and flying sparks increased again. This effect was further intensified by the fact that the metal vapor could not spread unhindered in all directions, but first spread out of the kerf solely upward.

**Figure 5:**
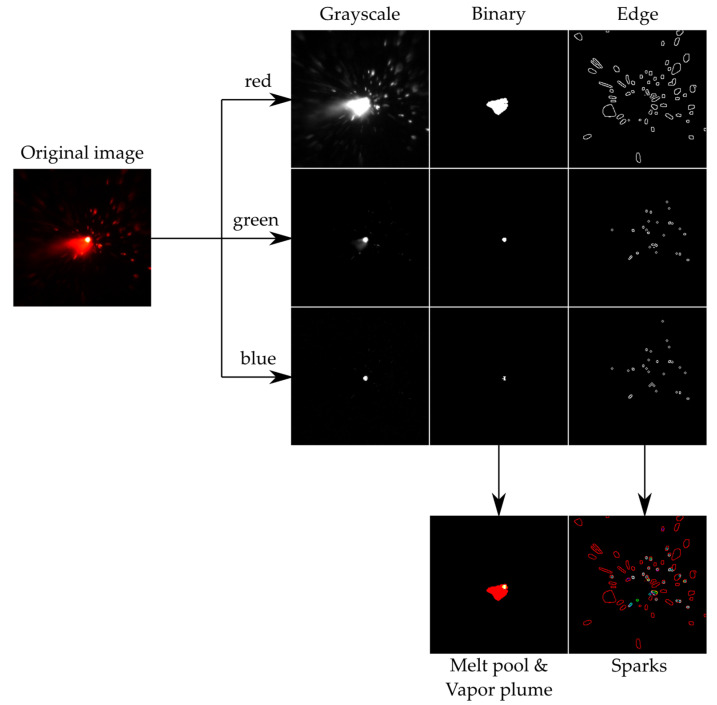
Illustration of the individual image processing steps. The original image and individual color channels of the camera, the binarized image, the edge detection, and the melt pool, vapor plume, and sparks are shown.

The following image processing steps were performed to obtain the information about the melt pool size, plume formation, and number of sparks, as illustrated in Figure 5. Each recorded image *I*(*u*,*v*,*c*) was a two-dimensional matrix with columns *u*, rows *v*, and a color channel *c*. The color information was digitized with a data depth of 8 bits for each color channel, i.e., the intensities for the red, green, and blue channels were available in 256 gradations. In the first step of this algorithm, the melt, the vapor plume, and sparks were separated from each other. The area of the melt was the hottest part in the kerf, radiating most intensively, appearing white in the center of the image. 

The melt area appeared white in the captured image because intensities in the red, green, and blue channels were detected and combined. Therefore, the hot area of the melt was particularly recognizable in the green and blue color channels. To determine the area of the melt, the image was first binarized for the green and blue color channel with the threshold value *th* = 245. The threshold value was chosen to be so high because the melt appeared white in the camera image due to the higher temperatures, as described previously.

(1) $$I_{bw}\left(u,v,c\right)=\{\begin{array}{c} 0\;\;\;\;for\;I\left(u,v,c\right)\leq th\\ 1\;\;\;for\;I\left(u,v,c\right)>th \end{array}.$$

From the binarized image, all adjacent connected white pixels were combined into one object. Pixels were connected if their borders were located next to each other. Two adjacent pixels were part of the same object if they were both white (or one) and connected along the horizontal or vertical direction. To obtain only the area of the melt without any sparks in the binary, the largest connected area was defined as the melting area. This was located in the center of the recorded image and, thus, in the kerf. Then, the sum of the largest object was set as the size of the melt pool for the green and blue color channels. The cooler vapor plume radiated much less intensely than the melt, glowed primarily in the red color channels, and was also positioned in the center of the captured image. Therefore, the area of the vapor plume was calculated from the red color channel. To determine the area of the vapor plume, the same steps were performed as for the calculation of the melt area, except that the threshold value was set to 75, since the cooler vapor plume radiated significantly less intensely. Lastly, the sum of the largest object was defined as the size of the melt pool for the green and blue color channels to *A_melt_*, and the size of the vapor plume was defined for the red color channel *A_plume_*.

(2) $$A\left(c\right)=\overset{u}{\underset{i=1}{\sum }}\overset{v}{\underset{j=1}{\sum }}I_{bw}\left(u+i,\;v+j,c\right).$$

(3) $$A_{plume}=max\left\{A\left(1\right)\right\},\;\;\;\;A_{melt\_g}=max\left\{A\left(2\right)\right\},\;\;\;\;A_{melt\_b}=max\left\{A\left(3\right)\right\}.$$

In parallel, the number of sparks was calculated. First, the images for the green and blue channel were binarized with *th* = 20. In the next step, the edges were extracted by a canny algorithm for each color channel. For edge detection, the image was first filtered with a Gaussian filter with the filter function *H*(*i*,*j*) for noise reduction, with a standard deviation of 0.5.

(4) $${I^{\prime }}_{bwS\;}\leftarrow \;\overset{i=1}{\underset{i=-1}{\sum }}\overset{j=1}{\underset{j=-1}{\sum }}I_{bwS}\left(u+i,v+j\right)\cdot H\left(i,j\right).$$

(5) $$H\left(i,j\right)=e^{-\frac{i^{2}+j^{2}}{2\sigma^{2}}}.$$

Then the gradient magnitude *G* was calculated as a directional filter response in the horizontal and vertical directions. With the absolute value of the gradient, all maxima were white; hence, each edge M and all zero values were black.

(6) $$\nabla G\left(u,v\right)=\left(\begin{array}{c} G_{x}\left(u,v\right)\\ G_{y}\left(u,v\right) \end{array}\right)=\left(\begin{array}{c} \frac{\partial I}{\partial x}\left(u,v\right)\\ \frac{\partial I}{\partial y}\left(u,v\right) \end{array}\right).$$

(7) $$M\left(u,v\right)=\sqrt{G_{x}^{2}+G_{y}^{2}}.$$

Edge selection *M_T_*(*u*,*v*) was achieved with a hysteresis thresholding method with two threshold values T1 and T2, where T1 = 5% and T2 = 9% of the maximum intensity. In the image, pixels larger than T2 were searched, and all adjacent pixels larger than T1 were marked as edges. Eventually, the area inside the edge was closed and set to white, while the number of sparks M_N_ was counted. Finally, the values of the melt pool size, plume size, and number of sparks for each color channel were stored for each individual frame, as well as the mean value and standard deviation for each cycle. 

## 3. Results and Discussion 

**Figure 6:**
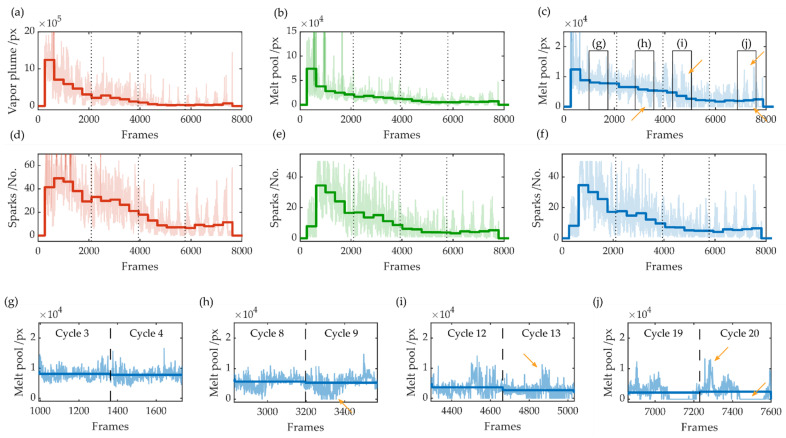
Signals of a complete cut with the vapor plume size (**a**), the melt pool sizes for the green channel (**b**) and blue channel (**c**), and the number of sparks for the respective color channels (**d**–**f**). The close-ups depict a magnified section of the signal of the melt pool size for the blue channel during the cutting process for a still incomplete cut (**g**), before a complete cut with occasional cut through (**h**), after a complete cut (**i**) and after too many cycles (**j**).

Figure 6 depicts an example of a measurement signal. Shown are the signal curves of the calculated values from the images processing step, for each frame, and the average value per cycle of the cutting contour. The size of the vapor plume *A_Plume_* and the melt pool sizes for the green channel *A_Melt_g_* and blue channel *A_Melt_b_* are shown in Figure 6a–c, while the numbers of sparks *M_N_* for the respective red, green, and blue channels are shown in Figure 6d–f. This cut was performed with a feed rate of 7 m/s, a laser power of 1.0 kW for 20 cycles, and a sheet thickness of 0.35 mm. At the selected sampling rate of 20 kfps, one frame took 0.05 ms, with 8000 frames accordingly taking 400 ms. One cycle took approximately 370 frames and, therefore, 18.5 ms, for the chosen geometry and feed rate. The categories, during the cutting process, for the first 10 cycles, after a complete cut and after too many cycles, for cycles 15–20, are shown in this measured signal. 

The average value for each cycle was used to decide after each contour pass if the workpiece was completely separated. For an overview and for better orientation, after five, 10, and 15 cycles, a vertical dotted line was plotted. In general, the size of the melt pool and the vapor plume decreased with increasing cycles. After the fifth cycle, the sheet was cut through for some positions. This is clearly visible, for example, in the blue channel of the melt during cycles 8 and 9, where single minima peaks can be seen for each cycle. After cycle 10, a complete cut occurred. Here, at cycles 12 and 13, the kerf of the contour was mostly opened toward the bottom, and the melt was partially expelled downward, with individual peaks where the material was molten. After too many cycles, for cycles 15–20, an increase in the measurement signal can again be observed. It is particularly noticeable that the signal of the melt pool was partly zero during one cycle, i.e., no measurable melt pool area can be detected in the video image, and peaks can be partly observed. The difference in the signals for a complete cut during cycles 12 and 13 and for too many cycles for cycles 19 and 20 can be explained by the propagation of the vapor capillary.

**Figure 7:**
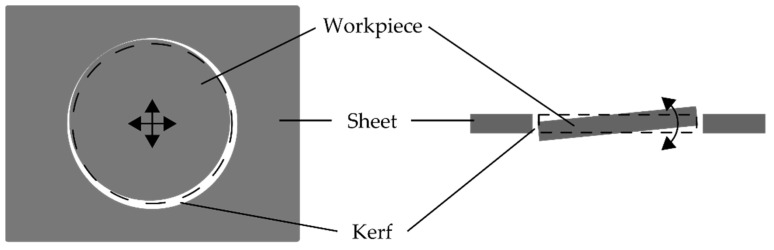
Schematic illustration for a possible displacement of the workpiece during too many cycles.

Figure 7 depicts a schematic illustration for a possible displacement of the workpiece for too many cycles. It is presumed that, during a complete cut, the metal vapor spreads not only upward, but also with in the kerf and under the sheet. This spread of metal vapor may also cause the workpiece to be moved and press against the sheet, or tilted before it falls out of the sheet. In this case, the kerf width was so large that the laser beam did not melt any material, i.e., no melt pool could be measured, or the kerf narrowed and material once again became molten. A similar behavior can be observed for the sparks. The number of sparks initially increased significantly with the second cycle and then decreased with increasing cycles.

**Figure 8:**
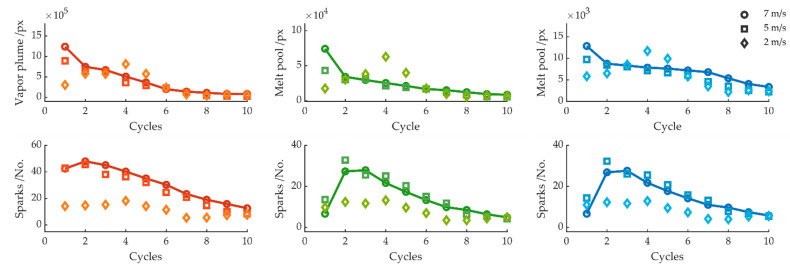
Average melt pool size and number of sparks per cycle for feed rates of 7, 5, and 2 m/s.

**Figure 9:**
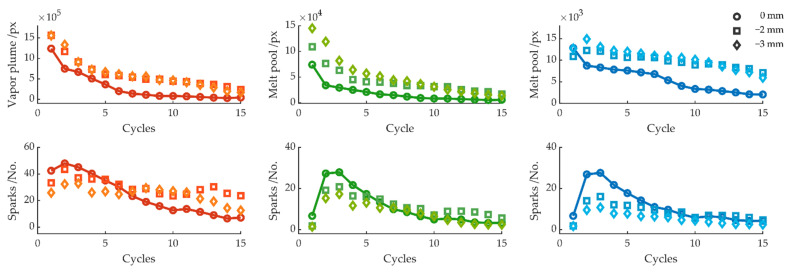
Average melt pool size and number of sparks per cycle for focus positions 0, −2, and −3 mm.

**Figure 10:**
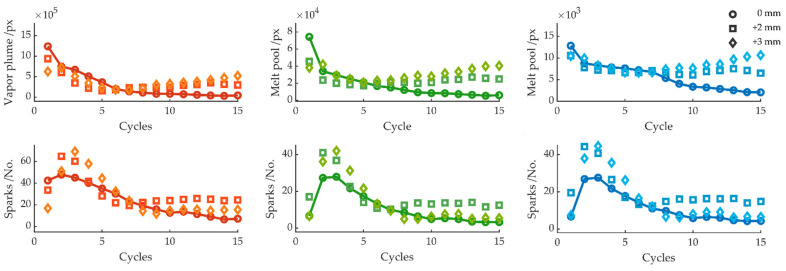
Average melt pool size and number of sparks per cycle for focus positions 0, +2, and +3 mm.

**Figure 11:**
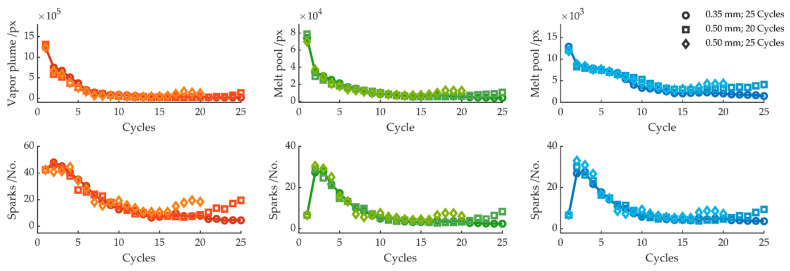
Average melt pool size and number of sparks per cycle for sheet thicknesses 0.35 and 0.50 mm.

Figure 8, Figure 9, Figure 10 and Figure 11 summarize the variation of the melt pool size, vapor plume, and sparks as a function of the feed rate, laser focus, and sheet thickness. The average value for each cycle is shown on the left side for the red color channel, in the center for the green color channel, and on the right side for the blue color channel. As a reference value for all parameter variations, the cutting experiment with a feed rate of 7 m/s, 1 kW laser power, and sheet thickness of 0.35 mm is compared. The best edge quality could be achieved with these cutting parameters. After 10 cycles, the workpiece was detached from the sheet and fell out immediately after the laser turned off. Figure 8 compares the melt pool and spark behavior for feed rates of 7, 5, and 2 m/s. When the feed rate was reduced to 5 m/s with otherwise unchanged parameters, no significant difference can be seen in the measured signals. Likewise, the area of the vapor plume and melt pool, as well as the number of sparks, decreased with increasing number of cycles until, after 10 cycles, a complete cut through occurred and the workpiece could be detached from the sheet. A slightly smaller melt pool size for a feed rate of 5 m/s can only be observed during the first cycle. A further reduction in the feed rate led to a wider kerf [42] but shorter melt pool length [43] from the top view. A considerably smaller melt pool area can be observed at cycle 1 and 2, increasing at cycle 3–6, which can be identified as a rewelding by the increasing area of the melt pool size, before the melt pool size finally decreased for the remaining cycles. However, for feed rates of 2 m/s and lower, the workpiece was firmly resealed to the sheet.

Figure 9 and Figure 10 show the signals with a negative and positive shift of the laser focus. Upon a focus shift, the absorbed area of the laser on the workpiece surface increased. As seen in Figure 9, for a negative focus shift, both the plume and the melt pool size were significant larger as compared to a complete cut. A lower number of sparks can be observed during the first five cycles. For the green and blue color channels, the number of sparks approached the refence cut after seven cycles, whereas the number of sparks increased significantly for the red color channel. For a positive focus shift, a noticeable increase for the melt pool area can also be seen, particularly clear from cycle 8 onward. The number of sparks significantly increased for the second and third cycles, dropped during cycle 5, and also approached the reference cut. From cycle 9, the number of sparks increased again, especially for a positive focus shift of +2 mm. In both cases, the kerf was sealed, and a complete cut through was not achievable. 

Figure 11 depicts the melt pool area, plume size, and number of sparks for cuts in a 0.50 mm thick sheet for 20 and 25 cycles. Compared to the sheet thickness of 0.35 mm, no difference can be found in the signal or camera image for cycles 1–15. For the sheet thickness of 0.5 mm, a decrease in the melt pool area and number of sparks can also be detected. However, the removal rate was too low for the thicker sheet; thus, after 15 cycles, a complete cut was not achieved, and the bottom edge of the sheet was still united. For further cycles, the signal showed an increase in melt pool size and consequently a rewelding of the kerf. It is worth mentioning that, although the workpiece was welded to the sheet and, therefore, did not fall out immediately after the laser turned off, it was possible to break the workpiece apart from the sheet. This is already indicated by a small variation in the measured signal. 

**Figure 12:**
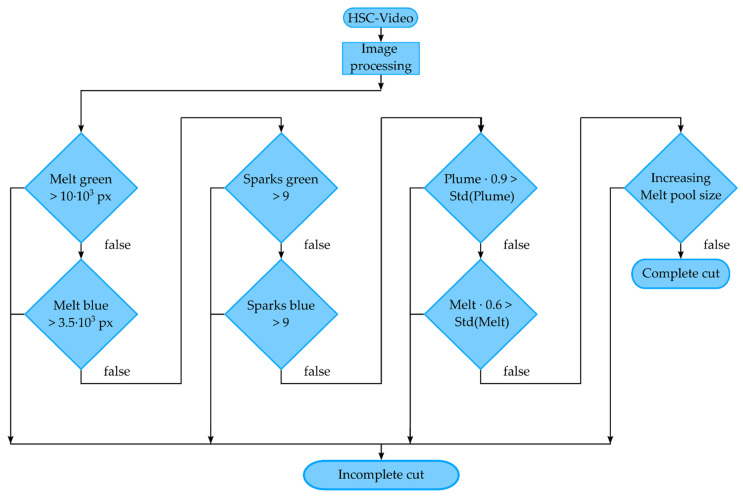
Flowchart of the algorithm to detect complete cuts.

A flowchart describing the cutting fault detection algorithm is illustrated in Figure 12. After the image processing section, the subsequent decisions to determine between a complete cut and an incomplete cut are processed after each cycle. The threshold value for the detection depends on the exposure time of the camera, as well as on the binarization method of the image process. The algorithm for detecting cutting error was first tested with different threshold values on eight cutting samples, four complete and four incomplete cuts each, and then applied to all 45 cutting samples. The first step is to check whether the blue and green melt pool sizes are larger than a selected threshold value, according to the experimental results (see Figure 8, Figure 9, Figure 10 and Figure 11). Concretely, a threshold greater than 10 × 10^3^ pixels was selected for the green melt pool area, while a threshold greater than 3.5 × 10^3^ pixels was selected for the blue melt pool area. This applies, for example, during the cutting process before a complete cut through. As soon as a complete cut occurs, this threshold value for the melt pool size is underrun, since the melt can now be ejected underneath the sheet. In the next step, the sparks are examined. Here, an experimental-based threshold value also determines whether more than nine sparks were detected for the green and blue color channel. This is the case during the cutting process, before a complete cut occurs, where sparks are ejected upward, since, even during a cut, not all sparks are expelled under the sheet, but sporadically ejected upward. Accordingly, for the case when the bottom edge of the sheet rejoins, by burr formation or rewelding of the kerf, sparks are no longer expelled downward, but evaporate toward the top side. With this preselection, 89% of all incomplete cuts can already be detected. The subsequent decisions refine the algorithm to detect special cases of incomplete cuts. In the next step, the standard deviation and mean value of the plume and melt pool area are compared. This was applied for cuts in the 0.50 mm thick sheet. Here, after 10–15 cycles, a small melt pool size and a low number of sparks were measured before burr formation and rewelding sealed the kerf again. In this case, the workpiece could only be broken out from the sheet. This behavior can be recognized in the measured signal by the fact that there were no negative peaks for each cycle (as described in Figure 6), but a constant noise in the measured signal, resulting in a low standard deviation. Finally, it is checked whether the melt pool size increases for the first three cycles. This is the case for low feed rates. Here, a small melt pool size was also measured, which increased with each cycle, resulting in a sealed kerf (see Figure 8). The algorithm described here provides the best detection probability, since all complete and incomplete cuts are detected. 

## 4. Conclusions

A monitoring system based on a high-speed camera for remote laser cutting and a specifically designed image processing algorithm were demonstrated. The possibility of in situ monitoring with direct observation of the emitted secondary process radiation from the process zone in the visible spectral range for scan head-based fiber laser remote processing without external illumination highlights the advantageous features of this approach. The images were taken with a sample rate of 20 × 10^3^ fps and an exposure time of 20 µs with a size of 256 × 256 pixels. The captured images were binarized with the edges being extracted to subsequently determine the size of the melt pool and the number of ejected sparks on the basis of different thresholds. The dependences of the feed rate, number of cycles, laser focus position, and sheet thickness on the area of the melt pool size and spark emissions from the top view were shown, allowing the definition of threshold values for the algorithm. Incomplete cuts caused by an insufficient number of cycles, too low feed rate, or a laser focus shift can successfully be detected, and the evaluation algorithm allows distinguishing between incomplete and complete cuts for remote laser cutting. 

## Data Availability

Not applicable.
